# Comparing the efficacy and safety of minimally invasive biportal endoscopic spine surgery versus conventional microscopic discectomy in single-level lumbar herniated intervertebral disc (ENDO-BH Trial): a multicenter, prospective, randomized controlled equivalence trial study protocol

**DOI:** 10.1186/s13063-022-06094-2

**Published:** 2022-02-22

**Authors:** Sang-Min Park, Kwang-Sup Song, Ho-Joong Kim, Si-Young Park, Taewook Kang, Min-Seok Kang, Dong Hwa Heo, Choon Keun Park, Dong-Geun Lee, Jin Sub Hwang, Jae-Won Jang, Jun Young Kim, Jin-Sung Kim, Hong-Jae Lee, Ki-Han You, Hyun-Jin Park

**Affiliations:** 1 Spine Center and Department of Orthopaedic Surgery, Seoul National University College of Medicine and Seoul National University Bundang Hospital, Seongnam, South Korea; 2 Department of Orthopaedic Surgery, Chung-Ang University, College of Medicine, Seoul, South Korea; 3 Department of Orthopaedics, Korea University College of Medicine, Anam Hospital, Seoul, South Korea; 4 Department of Orthopedic Surgery, Endoscopic Spine Surgery Center, Bumin Hospital, Seoul, South Korea; 5 Department of Neurosurgery, Endoscopic Spine Surgery Center, Seoul Bumin Hospital, Seoul, South Korea; 6 Department of Neurosurgery, The Leon Wiltse Memorial Hospital, Suwon, South Korea; 7 Department of Neurosurgery, Seoul St. Mary’s Hospital, The Catholic University of Korea, Seoul, South Korea; 8 Department of Neurosurgery, Daejeon St. Mary’s Hospital, The Catholic University of Korea, Seoul, South Korea; 9 Department of Orthopedic Surgery, Spine Center, Kangnam Sacred Heart Hospital, Hallym University College of Medicine, 1, Singil-ro, Yeongdeungpo-gu, Seoul, 07441 South Korea

**Keywords:** Lumbar disc herniation, Biportal endoscopic lumbar discectomy, Microscopic lumbar discectomy, Randomized controlled trial

## Abstract

**Background:**

Biportal endoscopic surgery has recently been performed in lumbar discectomy, with advantages over conventional surgery, such as less skin scarring and muscle damage. However, the clinical results have not been established. Although previous studies reported no difference between the biportal endoscopic and microscopic discectomy clinical results, the evidence was weak. Therefore, this study aims to evaluate the efficacy and safety of the biportal endoscopic discectomy versus the microscopic discectomy.

**Methods:**

This prospective multicenter randomized controlled equivalence trial is designed to compare the efficacy and safety outcomes of patients who underwent lumbar discectomy using biportal endoscopy or microscopy. We will include 100 participants (50 per group) with a lumbar herniated disc. The primary outcome will be the Oswestry Disability Index (ODI) score 12 months after surgery based on a modified intention-to-treat strategy. The secondary outcomes will include the visual analog scale score for low back and lower extremity radiating pain, the ODI score, the Euro-Qol-5-Dimensions score, surgery satisfaction, walking time, postoperative return to daily life period, postoperative surgical scar, and surgery-related variables, such as postoperative drainage, operation time, admission duration, postoperative creatine kinase, and implementation status of conversion to open surgery. Radiographic outcomes will also be analyzed using magnetic resonance imaging (MRI) or computed tomography (CT) and simple radiographs. Safety will be assessed by evaluating all adverse and severe adverse events and surgery-related effects. The participants will be assessed by a blinded assessor before surgery (baseline) and 2 weeks and 3, 6, and 12 months after surgery.

**Discussion:**

This trial will be the first prospective, multicenter, randomized controlled trial to analyze the efficacy and safety of biportal endoscopic discectomy in lumbar herniated disc.

This trial is designed for evaluating the equivalence of the results between biportal endoscopic and microscopic discectomy including adequate sample size, blinded analyses, and prospective registration to reduce bias. This trial will provide enough data on the effectiveness and safety of biportal endoscopic surgery and will be an important study that allows clear conclusions.

**Trial registration:**

Clinical Research Information Service (cris.nih.go.kr.) (KCT0006191). Registered on 27 March 2021

## Background

Discectomy for herniated discs is the most common method for resolving a patient’s symptoms [1, 2]. Currently, microscopic discectomy, a minimally invasive surgery, is performed to address the problems of conventional open discectomy [3]. As a representative method, microscopic discectomy, which uses a tubular retractor and an endoscope, is the most commonly used minimally invasive surgery method [1]. Minimally invasive surgery has many advantages over conventional methods, and reports indicate that the clinical results do not differ from conventional methods [3, 4]. A full-endoscopic discectomy (uniportal) is a minimally invasive surgery with a single very small incision. According to a randomized controlled trial by Gibson JNA et al., full-endoscopic discectomy showed similar functional improvement compared to microscopic discectomy, and it showed reduced length of hospital stay and less leg pain at 2 years after surgery [5]. However, the procedure is difficult to learn, has a narrow field of view and a long operation time, and may cause problems, such as insufficient discectomy [6].

Recently, the biportal endoscopic spine surgery was developed [7–13]. This surgical technique uses two small skin incisions, called portals, to access the surgical site, minimizing the damage to normal structures. Consequently, there are fewer post-surgical complications, such as postoperative pain and muscle damage. Additionally, arthroscopic instruments for knee and shoulder joint surgery can be used, eliminating the need to purchase additional equipment. Spinal instruments familiar to the spine surgeon, such as Kerrison punches and pituitary rongeurs, can also be used.

There are several retrospective clinical reports of biportal endoscopic discectomy [7, 14, 15]. A previous study reported that the clinical feasibility and results of biportal endoscopic discectomy were similar to conventional discectomy. However, the evidence suggesting advantages to biportal endoscopic discectomy was weak owing to the small number of patients, and the study was retrospective. Therefore, a randomized controlled trial (RCT) is warranted. This multicenter, prospective RCT will compare the outcomes of biportal endoscopic versus microscopic discectomy. We hypothesize that the efficacy and safety of biportal endoscopic discectomy and microscopic discectomy in the lumbar spine will be similar.

## Methods/design

### Trial design

The design and protocol of this multicenter, assessor-blind, prospective, parallel randomized controlled equivalence trial were approved by the institutional review board of participating hospitals in Korea. Participants will be randomly allocated 1:1 to either active intervention group or control intervention group. Participants randomized to active intervention group will undergo biportal endoscopic discectomy. And participants randomized to the control intervention group will undergo microscopic discectomy. Participants will visit the hospital for a minimum of 12 months at 2 weeks, 3 months, 6 months, and 12 months after surgery to evaluate the participant’s outcome.

### Participant population

One hundred adults aged 20–80 years with radiating pain in the lower extremities will be recruited across six hospitals. Enrollment eligibility will be determined based on the inclusion and exclusion criteria.

### Inclusion criteria

- Aged 20–80 years old
- Lumbar herniated intervertebral disc disease at one level
- Radiating pain to the lower extremities (visual analog scale [VAS] score > 4)
- Able to understand and consent to the research
- Willing to participate and to comply with our proposed follow-up protocol

### Exclusion criteria

- Spondylolisthesis (Meyer grade ≥ II)
- Spinal stenosis more than moderate degree (Schizas classification ≥ grade B) [16]
- A history of lumbar spinal surgery at the same level
- Degenerative lumbar scoliosis (Cobb angle > 20°)
- Other spinal diseases (e.g., ankylosing spondylitis, spine tumor, fracture, or neurologic disorders)
- Psychological disorders (e.g., dementia, intellectual disability, or drug abuse)
- Other disorders which the surgeon considers inappropriate for participation

### Recruitment

Participants will be recruited from those who decided to perform a one-level discectomy for lumbar disc herniation in each hospital; subjects will not be recruited by social media. Potential participants will be screened by the researcher to determine participation eligibility, and eligible participants will undergo baseline testing with a blind assessor. All participants will receive a baseline test and outcome assessment after providing written consent to the researcher.

### Randomization and follow-ups

Participants will be randomized into either the control (microscopy) or intervention (biportal) group at a 1:1 ratio, following a computer-generated randomization list prepared by a researcher with block sizes of four. The randomization lists will be incorporated into a web-based eCRF platform (iCReaT; internet-based clinical research and trial, icreat.nih.go.kr) accessible to authorized researchers. The randomization process will be processed independently at each hospital and completed by the researchers. Allocation will be concealed in opaque envelopes numbered consecutively and presented to the surgeon immediately before surgery.

**Fig. 1:**
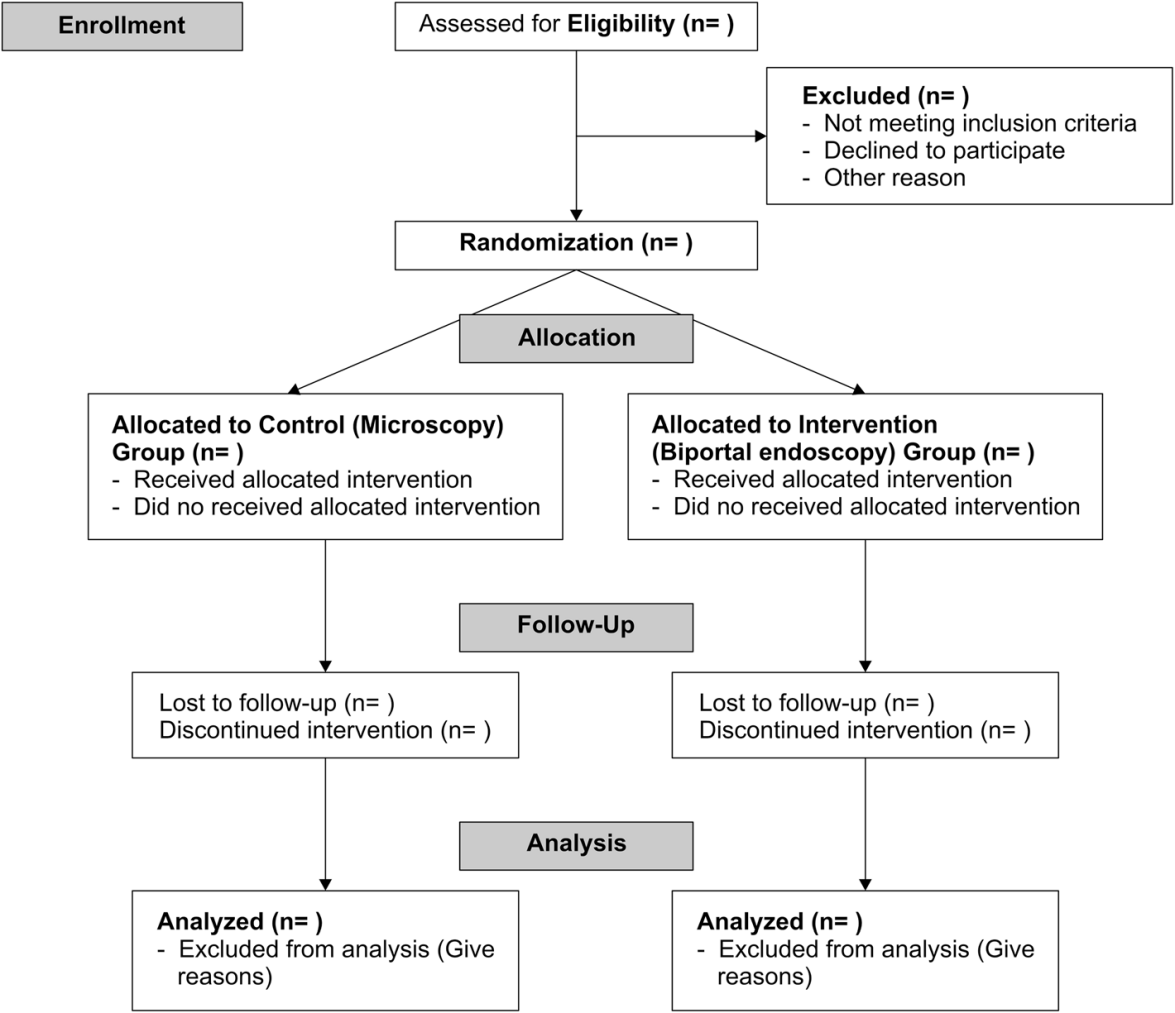
CONSORT study flow diagram of this study protocol

Participants will visit the hospital for a minimum of 12 months at 2 weeks, 3 months, 6 months, and 12 months after surgery to evaluate the participant’s outcome. The participant’s primary and secondary outcomes will be collected by an independent researcher during in-hospital visits but can be evaluated over the phone if unavoidable circumstances arise (Fig. 1).

The end of this trial is the date of the 12-month follow-up visit of the last participant. At the end of the trial, participants will resume standard practice of care according to the hospital’s routine follow-up schedule after discectomy.

### Blinding

This is a single (assessor) blind trial comparing biportal endoscopic discectomy with standard treatment (microscopic discectomy); the participants and surgeons will know what surgery they have undergone. Therefore, only the assessor will be blinded, and the blinded outcome assessor will measure and collect all outcomes before and after surgery, with a single-blind in each hospital. Each assessor will attend a training session prior to data collection to ensure consistency across hospitals. The outcome assessor will provide a detailed justification if unblinding occurs.

### Interventions

#### Active intervention: biportal endoscopy

**Fig. 2:**
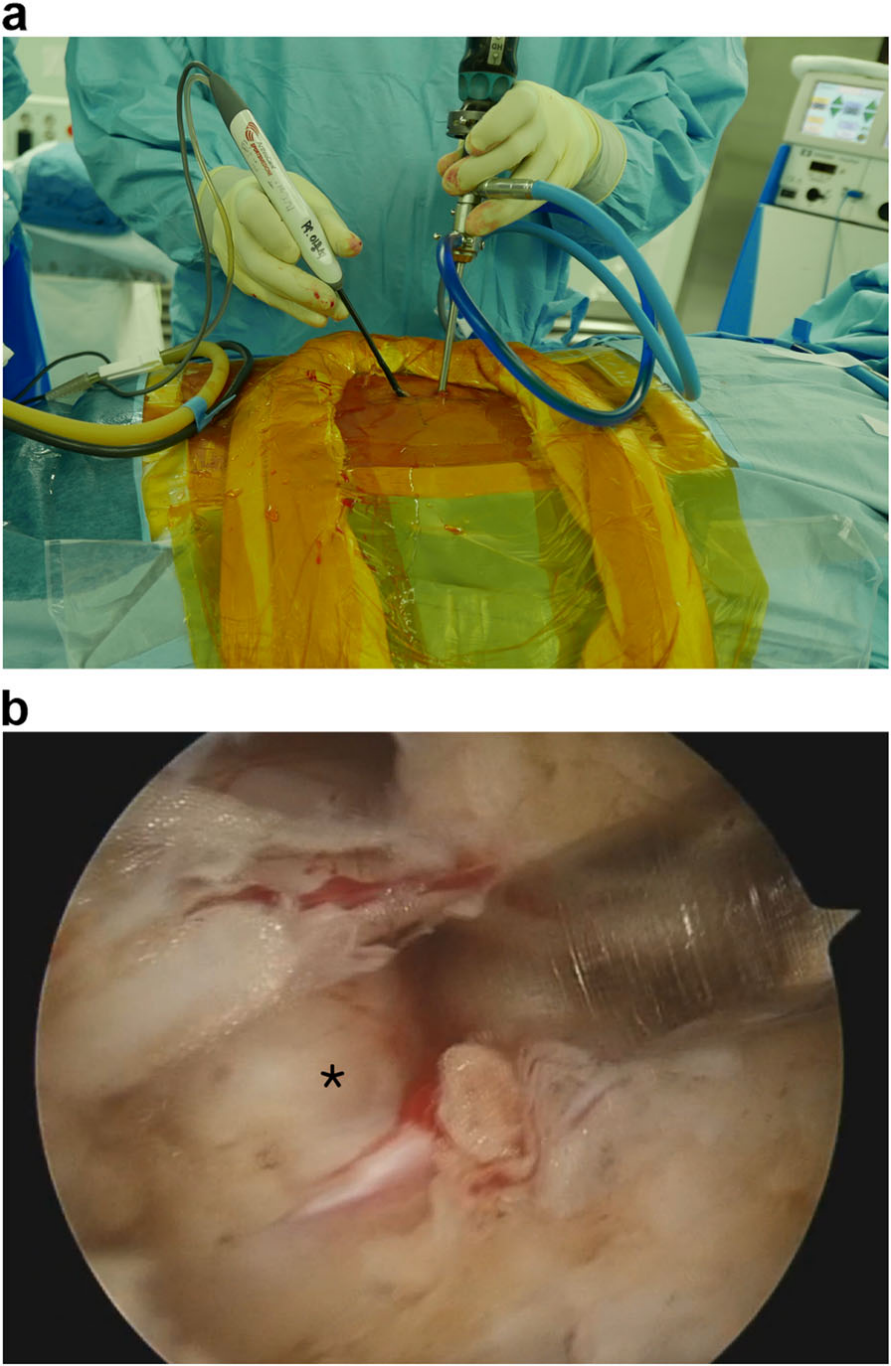
**A** Operative field of biportal endoscopic spine surgery in right-handed surgeon. **B** Intraoperative endoscopic view showed extruded disc(*)compressing nerve root

The biportal endoscopic discectomy has been previously described in several studies [9, 14, 15]. This surgical technique is similar to microscopic discectomy except that it involves making two portals. Therefore, the most important point of this surgical technique is creating a viewing portal for the camera and a working portal for the spinal instrument, which provides working space. The portal position starts from 0.5 to 1 cm lateral to the spinous process. With a right-handed surgeon and left-side approach as a reference, the working portal makes an incision about 1 cm below the lamina. The viewing portal is made vertically 1 cm proximal to the working portal with a 7-mm incision. When approached from the right side, the viewing portal is made vertically 1 cm distally to the working portal with a 7-mm incision. After creating the two portals, the paraspinal muscles will be detached from the lamina using a narrow Cobb elevator to ensure adequate working space. A 4-mm, 30° arthroscope is inserted through the viewing portal under saline irrigation with a pressure of 30-40 mm Hg. Surgery is performed by inserting the spinal surgical instruments, such as bipolar radiofrequency cauterization, burrs, Kerrison punches, and pituitary rongeurs, through the working portal. The frazzle muscle and soft tissue are removed using a shaver and bipolar radiofrequency cauterization. After the working space is created, the discectomy is performed in the same way as the microscopic discectomy (Fig. 2).

#### Control intervention: microscopic surgery

The microscopic discectomy procedure is a commonly used surgical technique for patients with a herniated disc. Briefly, the surgery level is checked by fluoroscopy, and a 3-cm midline incision is made. Following the skin incision, the paraspinal muscle is detached from the spinous process and the lamina using a Cobb elevator and towed with a Taylor retractor. Laminotomy is performed using burr and Kerrison punches. After removing a partial ligamentum flavum under the lamina, the dura and root are checked for discectomy. The root is retracted, and the disc is removed below the root, following which a check for any remnant disc is performed, and the operation is completed.

### Outcome measures

#### Primary outcome

The primary outcome will be the biportal endoscopic discectomy efficacy in the lumbar herniated disc. The efficacy will be determined by the Oswestry Disability Index (ODI) score [17]. The primary outcome is the ODI score 12 months after surgery between the two groups. The ODI is the most commonly used outcome measure questionnaire for lumbar disabilities in a hospital setting. This questionnaire is designed to evaluate various daily life activities, divided into ten sections. Each section is scored on a scale of 0-5, with a score of 5 representing the greatest disability. The ODI score is the summed score divided by the total possible score and expressed as a percentage (i.e., multiplied by 100). For all unanswered questions, the total possible score is reduced by 5. If the patient marks more than one statement in a question, the statement with the highest score is recorded as an indication of the actual disability.

#### Secondary outcome

The secondary outcomes are the clinical outcomes, radiographic outcomes, and adverse events. Clinical outcomes include (1) low back pain and lower extremities radiating pain, measured based on the VAS pain score, which ranges from zero (i.e., “none”) to 10 (i.e., “severe pain”), (2) quality of life (QOL), measured by the EuroQol-5-dimension-5-level [EQ-5D] questionnaire, which contains five questions with five responses for each question, and the total score is converted into the final EQ-5D value, ranging from 0.000 to 1.000; higher scores indicate a better QOL [18], (3) surgery satisfaction, (4) walking time, (5) the postoperative return to daily life period, (6) postoperative surgical scarring, measured by the Patient and Observer Scar Assessment Scale [POSAS] patient scale 2.0, containing six parameters with 10-point scoring system; 6 represents normal skin, and 60 represents the worst scar imaginable, and (7) surgery-related variables, such as postoperative drainage (mL), operation time (minutes), admission duration (hours), postoperative creatine kinase, and implementation status of conversion to open surgery. The radiographic outcomes include (1) the degree of disc removal and facet joint injury, measured using postoperative MRI or CT and (2) other radiographic complications, measured using simple radiographs during the follow-up period. Safety will be assessed by evaluating all adverse and severe adverse events and surgery-related effects (reported to the appropriate institution, as needed). This will be reported by the participant to the surgeon or the assessor and recorded in the electronic database.

Baseline radiographs will be made in the anteroposterior, lateral, flexion, and extension view, and spondylolisthesis and segmental instability at the surgery level will be scored. A preoperative spine MRI will be systematically checked. Based on the lumbar disc nomenclature of David F. Fardon et al., extruded, sequestrated, and migrated disc will be classified according to disc type, and central canal zone, subarticular zone will be classified according to location [19]. The degree of annular defect of the disc will be evaluated through Carragee classification [20]. Also, disc herniation side and severity of the canal compromise in the axial plane will be checked. Patient-reported outcomes will be collected from the participants at baseline and at 2 weeks and 3, 6, and 12 months after surgery. These outcomes will be collected by the blinded assessor and recorded in the eCRF system (Table 1).

**Table 1:** Evaluation schedule

Visit type	Screening	Operation/treatment	Follow-up			
Visit	1	2	3	4	5	6
Visit week	− 4~0 weeks	0–2 day	2 weeks	12 weeks	24 weeks	52 weeks
			± 5 days	± 4 weeks	± 8 weeks	± 8 weeks
Informed consent	■					
Demographics*	■					
Inclusion/exclusion	■					
Randomization		■				
Operation		■				
MRI (or CT)†	■	■				
Simple radiographs	■		■	■	■	■
ODI	■		■	■	■	■
EQ-5D-5L	■		■	■	■	■
VAS	■		■	■	■	■
POSAS				■	■	■
Other surveys^‡^			■	■	■	■
Adverse events		■	■	■	■	■

*MRI* magnetic resonance imaging, *CT* computed tomography, *ODI* Oswestry Disability Index, *EQ-5D-5L* EuroQol 5 Dimension 5 level, *VAS* visual analog scale, *POSAS* Patient and Observer Scar Assessment Scale

*Baseline patients’ characteristics including past medical/surgical history, physical examination, and laboratory tests

^†^CT scan is possible when MRI cannot be taken

^‡^Including surgery satisfaction, walking time, postoperative return to daily life period

#### Statistical analysis

All statistical analyses will be conducted using Stata/MP 15.1 (StataCorp LLC, College Station, TX). A two-sided *P*-value < 0.05 will be considered statistically significant. The Shapiro-Wilk test will be used to evaluate the distribution of the collected data. Normally distributed continuous variables will be presented as the mean and standard deviation (SD), whereas non-normally distributed variables will be presented as the median and interquartile range. Categorical variables will be presented as numbers and percentages (%).

Both modified intention-to-treat (mITT) and per-protocol analyses will be performed. The mITT strategy will be the main analysis and indicates that participants are analyzed on whether they underwent a randomly assigned surgery (to avoid the effects of crossover and dropout, which may break the random assignment to the treatment groups). Excluded participants before and after surgery will be excluded from the analysis, and participants exceeding the calculated sample size will not be recruited.

The surgical intervention effect 12 months after surgery will be assessed by the ODI scores and a two-sided 95% confidence interval (CI) as the primary outcome and then compared between the groups. Biportal endoscopic discectomy will be considered equivalent to microscopic discectomy if the upper and lower limit of 95% CI of the ODI score at 12 months is limited to within the pre-defined equivalence limit of 12.8 points. To analyze the serial effect on secondary clinical outcomes (i.e., VAS pain scores for the back and lower extremities and the ODI, EQ-5D, and POSAS score), a linear repeated-measures mixed model will be used. Time will be analyzed as a categorical variable (2 weeks and 3, 6, and 12 months) and include the intervention-time interaction to analyze the effects of surgery during the follow-up periods. Inter-group differences during the 12-months will also be analyzed using a linear repeated-measures mixed model, controlling for baseline and follow-up time points as categorical variables. Other clinical, radiographic outcomes and adverse effects between two groups will be analyzed using Student’s *t*-test for continuous variables and the chi-square test for categorical variables.

#### Data management

Anonymized participant data will be entered into the electronic research database (internet-based Clinical Research and Trial management system, iCReaT). The iCReaT system was created by the government and designed so that investigators and researchers can securely and directly input patient research data. This database is equipped with several security devices to protect data and prevent unauthorized access and information disclosure using a web-based encryption system. All data is assigned to each patient’s study number and processed anonymously. This study number is provided to the researchers and investigators only. The anonymized outcome data is only available and accessible to the leading researchers, investigators, and statistical analysts.

Designated monitoring researchers, delegated by the primary monitoring researcher, will carry out checks to determine if the clinical trial is being conducted per the Korea Good Clinical Practice and related regulations and according to the clinical trial protocol. Monitoring will be conducted in parallel with on-site (i.e., institution visits) and in-house monitoring (i.e., through the electronic data capture system). On-site monitoring will be conducted ten times in total, including the site initiation visit and the site close-out visit. In this study, the dataset from the electronic case report form (e-CRF) system will be used, and the clinical research associate from the contract research organization will monitor the completeness and accuracy of the data collected by the researchers. Data with problems or questions will be sent to the researcher through the e-CRF system query function to check and correct the data. The modified database will be saved, and all changes to the database will be recorded. All research documents will be coded and stored separately and managed so that personal identification through the data is impossible.

#### Safety reporting

Regarding the biportal endoscopic discectomy and microscopic discectomy to be performed in this trial, there are no additional complications due to participation of this trial. Complications of general spinal surgery, such as bleeding, infection, dural tear, nerve root damage, bowel and bladder incontinence, pneumonia, deep vein thrombosis, and requirement for revision surgery should be fully explained before the start of the trial.

If unexpected adverse events occur during the course of this trial, the researcher should immediately report it to the institutional review board of the relevant hospital, the principal investigator, and the sponsor. Also, if the participant needs treatment, first aid is implemented as soon as possible. Adverse reactions that occurred during the trial will be followed up until symptoms resolved or stabilized.

Any harm from the intervention that occurred to the participants during this trial will be divided into adverse events (AEs), serious adverse events (SAEs), and suspected unexpected serious adverse reactions (SUSARs). The chart is drawn up in the order of symptom occurrence according to the severity, and participants check it when they visit the outpatient clinic. Adverse reactions should be described in detail in the special form of the case report form regarding symptoms, duration, severity, causal relationship with intervention, additional treatment, results of adverse reactions, and severity.

#### Sample size

This trial will recruit 100 participants (50 participants per group) to confirm the primary outcome equivalence between biportal endoscopic and microscopic discectomy. According to a previous report [21], the minimal clinically important ODI difference was 12.8, and in the previous study [22], the standard deviation of the ODI value 1 year after endoscopic discectomy was 17.1. Assuming that the equivalence limit is 12.8, alpha = 0.05, power = 0.90, two-sided 95% CI, and the follow-up loss = 20%, 50 participants are needed for each group. Power Analysis and Sample Size software version 15 (NCSS, Kaysville, UT, USA) was used for calculating the sample size.

#### Ethics and dissemination

The design and protocol of this multicenter, assessor-blind, prospective, RCT have been approved by the institutional review board of six hospitals (Seoul National University Bundang Hospital, B-2102/666-007; Hallym University Kangnam Sacred Heart Hospital, HKS202102023-HE002; Chung-Ang University Hospital, 2120-006-453; Korea University Anam Hospital, 2021AN0128; Wiltse Memorial Hospital, 2021-W01; Seoul Bumin Hospital, etc._21_003). All modifications that may affect the research data will be approved by the research ethics committee before implementation.

The study results will be submitted for peer-review publications. Additionally, electronic data will be anonymized and uploaded to an electronic database server (iCReaT) that supports limited access. Electronic data will not be made publicly available, and access to the data set will only be provided by the data management committee of the government’s research consortium.

## Discussion

Discectomy has developed over time. In 1977, Caspar et al. introduced microscopic discectomy, a less invasive method compared to conventional technique [23]. After the introduction of percutaneous posterolateral discectomy by Parvis Kambin in 1987 to reduce paraspinal muscle damage, percutaneous endoscopic lumbar discectomy (PELD) has been widely used, and this can be considered the beginning of endoscopic discectomy [24]. However, PELD has limitations in movement by docking an endoscope on the lesion, and it is not effective in nerve compression or stenosis caused by degenerative osteophytes. In 2013, Soliman first introduced the concept of biportal endoscopic surgery [25]. During this surgery, one can freely manipulate surgical instruments through two portals, secure a wider field of view, and remove the osteophyte by using a burr or osteotome more easily.

There have been some previous studies on the efficacy of biportal endoscopic discectomy [7, 14, 15]. In a multicenter, retrospective analysis of 141 patients with single-level lumbar disc herniation, biportal endoscopic discectomy yielded similar clinical outcomes, including pain control, functional disability, and patient satisfaction, compared to open microscopic discectomy and showed minimal estimated blood loss, shorter hospital stay, and less early postoperative back pain [8]. In addition, there are some studies that have reported that it can be applied even in complicated cases. Kang et al. reported that biportal endoscopic discectomy showed satisfactory clinical outcomes even in cases of high-grade migrated lumbar disc herniation [26]. Moreover, Kang et al. compared biportal endoscopy and open microscopy with revisional discectomy and reported that both techniques showed similar clinical outcomes at 1 year after surgery. The biportal endoscopic group showed faster pain relief, earlier functional recovery, and better patient satisfaction [13]. Thus, biportal endoscopic discectomy may be a feasible option.

However, studies on biportal endoscopic discectomy have limitations as retrospective studies, and an RCT has not yet been performed. This trial will be the first prospective, multicenter, RCT to analyze the efficacy and safety of biportal endoscopic discectomy in lumbar herniated disc. This trial is designed for evaluating the equivalence of the results between biportal endoscopic and microscopic discectomy including adequate sample size, blinded analyses, and prospective registration to reduce bias. This trial will provide enough data on the effectiveness and safety of biportal endoscopic surgery and will be an important study that allows clear conclusions.

## Trial status

Recruitment of participants started on July 20, 2021 and will be continued until the required number of participants will be enrolled. The estimated completion of recruitment date is July 2023, and estimated completion of final follow-up date is July 2024. We registered this trial on cris.nih.go.kr. (KCT0006191) and protocol version is “v1.1, 01 March 2021”

## Data Availability

The electronic database server (iCReaT) will not be publicly accessible. Access to the data set is provided only to the Data Management Committee of the Korean Government Research Consortium.

## Abbreviations

RCT: Randomized controlled trial
ODI: Oswestry Disability Index
QOL: Quality of life
MRI: Magnetic resonance imaging
CT: Computed tomography
mITT: modified intention-to-treat
CI: Confidence interval
AEs: Adverse events
SAEs: Serious adverse events
SUSAR: Suspected unexpected serious adverse reactions
e-CRF: Electronic case report form
